# Emicizumab prophylaxis vs immune tolerance induction in children with severe hemophilia A and inhibitors: a retrospective comparison of bleeding control, quality of life, and cost

**DOI:** 10.1016/j.rpth.2026.103425

**Published:** 2026-03-25

**Authors:** Zhengping Li, Qianqian Mao, Gang Li, Xiaoling Cheng, Yingzi Zhen, Guoqing Liu, Wanru Yao, Zekun Li, Jialu Zhang, Shuyue Dong, Di Ai, Zhenping Chen, Runhui Wu

**Affiliations:** 1 Hematology Department, Beijing Key Laboratory of Pediatric Hematology Oncology National Key Discipline of Pediatrics (Capital Medical University) Key Laboratory of Major Diseases in Children, Ministry of Education, Beijing Children's Hospital, National Center for Children's Health, Capital Medical University, Beijing, China; 2 Department of Clinical Laboratory Center National Key Discipline of Pediatrics (Capital Medical University) Key Laboratory of Major Diseases in Children, Ministry of Education, Beijing Children's Hospital, Capital Medical University, National Center for Children's Health, Beijing, China; 3 Department of Pharmacy, Beijing Children's Hospital, Capital Medical University, Beijing, China; 4 Cell and Gene Therapy Laboratory, Beijing Pediatric Research Institute, Beijing Children’s Hospital, Capital Medical University, National Center for Children's Health, Beijing, China

**Keywords:** Children, emicizumab, immune tolerance induction, inhibitors, severe hemophilia A

## Abstract

**Background:**

There is limited evidence directly comparing emicizumab (EMI) prophylaxis and immune tolerance induction (ITI) in children with severe hemophilia A and high-titer inhibitors (SHAcwHTI), particularly bleeding control, quality of life (QoL), and cost.

**Objectives:**

This study compared outcomes of EMI vs ITI in SHAcwHTI, focusing on bleeding rates, QoL, and costs.

**Methods:**

This single-center retrospective study enrolled SHA children (inhibitor titer ≥5 Bethesda Units/mL), receiving EMI or ITI from January 2020 to December 2024. EMI included loading (initial 4 weeks) and maintenance doses; ITI involved intermediate-dose (factor [F]VIII 100 IU/kg/d) or low-dose (FVIII 50 IU/kg once every other day). Outcomes included annualized bleeding rate (ABR), annualized joint bleeding rate (AJBR), Canadian Hemophilia Outcomes-Kids Life Assessment Tool scores, and medication costs.

**Results:**

Among 140 patients (24 in EMI group and 116 in ITI group [40 intermediate-dose ITI and 76 low-dose ITI]), EMI was associated with better bleeding control: median ABR and AJBR were 0 across all observation periods, significantly lower than ITI. For ITI, both ABR and AJBR declined over time but remained higher than those of EMI group. Target joint proportion decreased most markedly in patients receiving EMI (35.7%-0%; *P* = 0.002). EMI group also showed greater QoL improvement (mean change in parent proxy-reported Canadian Hemophilia Outcomes-Kids Life Assessment Tool scores: +26.6 vs +19.2 vs +19.1; *P* < .001) and lower medication costs (1993.4 vs 3703.4 vs 3656.3 US$/kg; *P* < .001).

**Conclusions:**

EMI prophylaxis was associated with improved bleeding control and QoL while reduced costs compared with ITI, offering a valuable option for SHAcwHTI, prioritizing immediate hemostasis over the long-term goal of inhibitor eradication, especially in resource-limited settings.

## Essentials

• There is lack of evidence comparing emicizumab (EMI) vs immune tolerance induction (ITI).
• This single-center retrospective study compares bleeding control, quality of life (QoL), and cost between EMI and ITI.
• Compared with ITI group, EMI group had better bleeding control and QoL and lower medication cost.
• Without considering inhibitors eradication, EMI may be a preferable option.

## Graphical abstract

**Figure:**
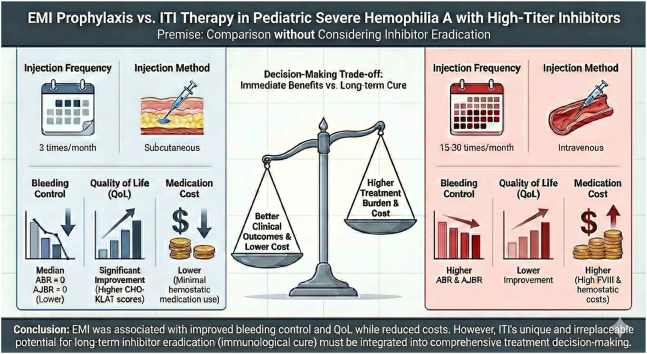

## 1 Introduction

The development of inhibitors is the most serious complication in severe hemophilia A (SHA), leading to significant morbidity and mortality due to treatment-resistant bleeding [1,2]. About two-thirds of the inhibitors are of high-titer (HTI, ≥5 Bethesda Units [BU]/mL). Inhibitors render factor replacement therapy ineffective [3]. Bypassing agents including activated prothrombin complex concentrate and recombinant factor (F)VIIa (rFVIIa) are used to achieve hemostasis in these patients; however, concerns about suboptimal efficacy and disease burden may remain [4].

The only effective treatment to eradicate inhibitors was immune tolerance induction (ITI), consisting of frequent high-dose FVIII to induce immune tolerance. Additionally, the international-ITI study reported that ITI regimens provided some degree of protection from intercurrent bleeding when the inhibitor has fallen to a low level [5]. However, ∼30% of patients fail to achieve tolerance despite prolonged treatment.

Emicizumab (EMI), a bispecific antibody mimicking FVIII cofactor function, bridges activated FIX and FX to restore hemostasis independent of inhibitor status [6]. EMI provides effective prophylaxis in the setting of an inhibitor, changing conversations around ITI. While the Atlanta protocol proposed combining low-dose ITI with EMI to simultaneously eradicate inhibitors and control bleedings [7], this approach remains unaffordable for resource-constrained regions.

This raises a critical question: Should patients with SHA with inhibitors prioritize ITI for inhibitor eradication, EMI for bleeding control, or a combination of both? In particular, in economically limited settings, the optimal treatment strategy remains uncertain. Therefore, this study specially compared the real-world effectiveness of EMI prophylaxis with that of ITI in SHA children with HTI (SHAcwHTI), focusing on 3 clinically relevant outcomes—bleeding control, quality of life (QoL), and medication costs. Notably, we intentionally excluded inhibitor eradication as an outcome measure, given that EMI does not target inhibitor clearance. This allowed for a direct comparison of the 2 therapies’ practical benefits in daily management.

## 2 Methods

### 2.1 Design

This single-center retrospective cohort study enrolled SHAcwHTI who received either EMI prophylaxis or ITI at Beijing Children’s Hospital, China, between January 2020 and December 2024, to compare the bleeding control, QoL, and medication cost. The study was approved by the ethics committee of Beijing Children’s Hospital ([2022]-E-166-Y). All research was conducted according to the principles outlined in the Declaration of Helsinki. ITI treatment includes low-dose (LD; FVIII 50 IU/kg once every other day) and intermediate-dose (MD; FVIII 100 IU/kg/d) [8], regardless of the kind of FVIII. EMI prophylaxis includes loading dosage for initial 4 weeks, followed by a maintenance dose thereafter. (See Section 2.4 for specific regimens.)

The primary outcome was bleeding control, in terms as annualized bleeding rate (ABR) and annualized joint bleeding rate (AJBR). Secondary outcome measures included the following: number and proportion of target joints, and zero bleeding proportion (ZBP) [9], scores of Canadian Hemophilia Outcomes-Kids Life Assessment Tool (CHO-KLAT) [10], number of injections per year; medication cost. (See Section 2.5 for specific definitions.)

### 2.2 Participants

#### 2.2.1 Inclusion criteria

Participants eligibility criteria included the following: (i) diagnosis of SHA ([FVIII clotting activity <1%] before inhibitor development) [11]; (ii) peak inhibitor titer of ≥5 BU/mL; (iii) received MD/LD-ITI treatment or received EMI prophylaxis regardless of with a history of ITI treatment.

#### 2.2.2 Exclusion criteria

Participant exclusion criteria included the following: (i) having an inherited or acquired bleeding disorder other than hemophilia A (HA); (ii) having concomitant immunologic disease; and (iii) are participating in or planning to participate in another interventional study.

### 2.3 Laboratory methods and inhibitor monitoring

FVIII clotting activity was determined using a 1-stage clotting assay. Inhibitors before and after EMI initiation were measured by the Bethesda assay, using 1-stage and chromogenic method, respectively [12]. During ITI, inhibitor titer was initially monitored every 1 to 2 weeks until a downward trend was evident after the initial peak from early repeated FVIII exposure and then monthly until the study end [13,14]. During EMI prophylaxis, patients visited a clinic once every 3 to 6 months according to bleeding control.

### 2.4 Treatment strategy

LD-ITI group was treated with FVIII 50 IU/kg once every other day and MD-ITI group with FVIII 100 IU/kg daily. The choice of recombinant FVIII or plasma-derived FVIII brand used for ITI was left to the discretion of the managing clinician.

Immunosuppressants (ISs) were added to patients receiving either ITI if the inhibitor titer (i) increased to ≥200 BU/mL during ITI [14]; (ii) increased continuously within 3 months after ITI start [15]; or (iii) decreased by <20% in any 6 months after ITI start [15]. ISs included rituximab 375 mg/m^2^/wk (maximum 600 mg) for 4 weeks, together with prednisone 2 mg/kg/d (maximum 60 mg) for 1 month, and then tapered over 6 weeks. Intravenous immunoglobulin (200 mg/kg monthly for 6 months) was administered to remedy the acquired IgG deficiency during this 6-month period after rituximab treatment for infection prevention.

Once the patient had achieved success (defined as negative inhibitor titer twice consecutively at least 4 weeks apart together with FVIII recovery ≥66% of expected), the FVIII dose would be reduced slowly to 25 to 30 IU/kg 3 times/wk for continuing prophylaxis [14].

#### 2.4.1 EMI prophylaxis group

All patients initiated their EMI prophylaxis regimen following a clinic visit. Patients were prescribed a standard loading dosage of EMI at 3.0 mg/kg weekly for 4 weeks, and maintenance EMI dosage at 1.5 mg/kg weekly, or 3.0 mg/kg every 2 weeks, or 6.0 mg/kg every 4 weeks. Although the patients were prescribed standard dosages, they often reduced their dosage by decreasing the amount per injection or altering the intervals between injections to lower costs during both the loading and maintenance periods.

#### 2.4.2 Control of breakthrough bleeding in ITI group

For ITI group, patients whose inhibitor titer was >2 BU/mL at the time of bleeding received rFVIIa, 90 μg/kg, repeated every 2 to 4 hours or a 4 factor prothrombin concentrate complex 40 to 50 IU/kg, repeated every 8 to 12 hours until the bleeding was controlled [16]. Patients whose inhibitor titer was <2 BU/mL at the time of bleeding received rFVIII, 20 to 50 IU/kg, repeated every 8 to 12 hours until the bleedings were controlled [17].

### 2.5 Outcome measures

#### 2.5.1 Observation periods definition

To facilitate comparative analysis between EMI and ITI groups, we defined the observation periods as follows:

(i) Phase S (success-equivalent phase)—for the ITI group, duration from treatment initiation to achievement of ITI success (defined as 2 consecutive negative inhibitor titers ≥4 weeks apart with FVIII recovery ≥66% of expected); for the EMI group, the average time to ITI success, derived from our ITI cohort (mean, 9.5 months), ensuring comparable observation windows.
(ii) Phase 1: 0 to 12 months (first year from treatment initiation)
(iii) Phase 2: 0 to 24 months (first 2 years from treatment initiation)
(iv) Phase 3: 0 to 36 months (first 3 years from treatment initiation)

#### 2.5.2 Bleeding and joint bleeding

The overall bleeding and joint bleeding were obtained through their routine bleeding records and verified at each follow-up visits.

(i) ABR and AJBR were calculated as follows: (number of bleedings/total number of days during the efficacy period) × 365.26 [12].
(ii) Joint bleeding [18]: an unusual sensation aura in the joint, in combination with any of the following: (a) increasing swelling or warmth of the skin over the joint; (b) increasing pain; or (c) progressive loss of range of motion or difficulty in using the limb compared with baseline.
(iii) Target joint [18]: Three or more spontaneous bleeding events into a single joint within a consecutive 6-month period. When ≤2 bleeding into the joint within a consecutive 12-month period, the joint is no longer considered a target joint.
(iv) ZBP: the proportion of zero bleeding during the observation period.

#### 2.5.3 Quality of life

First, patients’ QoL was estimated by the CHO-KLAT. Detailed information on these scoring scales can be found in previously reported studies [10]. CHO-KLAT reports were obtained at baseline (pretreatment) and posttreatment (≥1 year after initiation) during clinic visits. Second, the injection frequency was calculated by dividing the number of injections by phase S.

#### 2.5.4 Cost

Medication cost (per kilogram of body weight) was calculated based on the EMI/FVIII cost plus cost of PCC and rFVIIa for treatment of breakthrough bleeding during phase S. Costs of drugs were calculated based on the standardized national public procurement prices at our center. To ensure an objective comparison of the total economic burden on the health care system, this study used the total drug cost rather than the patients’ out-of-pocket expenses. This approach eliminates bias arising from differential insurance coverage policies (eg, FVIII is partially reimbursed while EMI is currently self-pay in China) and provides a consistent baseline for comparison.

### 2.6 Statistical analysis

The statistical analyses were performed using SPSS, version 26.0 (IBM Corp). The figure generation were performed using GraphPad Prism for Windows (version 9.1). Data were reported as median (IQR) and mean (SD). Categorical variables, expressed as frequencies and percentage values. Mann–Whitney U-test was used to evaluate the difference of ABR, AJBR, CHO-KLAT scores, and medication cost. Fisher exact chi-squared test was used to compare the ZBP and the proportion of target joint. The comparison of these indicators was conducted between baseline period and post-EMI/ITI treatment as well as between EMI group and ITI group. The *p* values <0.05 indicates a statistically significant difference. Since this was a retrospective study, the sample size was determined by the availability of eligible patients during the study period rather than an a priori power calculation.

## 3 Results

### 3.1 Study population and baseline clinical characteristics

Total 140 patients were included at median age at data analysis of 6.3 years. Among them, 116 (82.9%) patients received ITI treatment (40 in MD-ITI group; 76 in LD-ITI group) for median 2.4 years. Moreover, 24 (17.1%) patients received EMI prophylaxis for median 3.7 years, with median loading dose of 2.3 mg/kg/wk and maintaining dose of 5.4 mg/kg/mo. Among the 24 patients receiving EMI, 5 patients had a history of ITI (3 achieved ITI success; 1 had inhibitor titer increased to >0.6 BU/mL after success; and 1 remained inhibitor-titer positive for 24.8 months).

The historical peak titer pre-EMI/ITI were not significantly different between EMI group and ITI group (median, 28.9 vs 17.5 BU/mL; *P* = .276). After EMI/ITI treatment start, the peak titer was also similar between EMI group and ITI group (6.3 vs 21.7 BU/mL; *P* = .160) (Table 1). Before EMI/ITI treatment (baseline), both ABR (23.8 vs 25.1 vs 24.3; *P* > .05) and AJBR (14.7 vs 16.2 vs 15.9; *P* > .05) were similar among EMI, MD-ITI, and LD-ITI groups.

**Table 1:** Demographic, clinical, and laboratory characteristics of study participants by treatment arm.

Variables	Total	EMI prophylaxis	ITI			*P*^a^
			Total	MD-ITI	LD-ITI	
*n*	140	24	116	40	76	—
Hemophilia severity at baseline						
Severe	140 (100)	24 (100)	116 (100)	40 (100)	76 (100)	—
Age at analysis (y)	6.3 (4.2)	10.0 (5.0)	5.9 (3.5)	5.0 (4.2)	6.3 (3.6)	.619
Age at inhibitor-diagnosis (y)	2.2 (3.2)	1.8 (3.9)	2.3 (3.2)	2.5 (3.3)	2.3 (3.0)	.846
Initial inhibitor titer (BU/mL)	10.8 (30.2)	24.6 (36.8)	9.2 (25.6)	12.7 (27.2)	6.8 (20.7)	.759
Immediate titer pre-EMI/ITI (BU/mL)	14.2 (46.1)	13.8 (69.9)	14.4 (42.3)	18.4 (51.4)	10.4 (39.2)	.823
Historical peak titer pre-EMI/ITI (BU/mL)	24.0 (81.6)	28.9 (74.4)	17.5 (63.8)	24.0 (62.2)	16.0 (64.6)	.276
Peak titer post-EMI/ITI (BU/mL)	19.4 (107.1)	6.3 (20.3)	21.7 (146.6)	24.0 (118.2)	21.4 (161.9)	.160
FVIII gene variants^b^	113 (100)	23 (100)	90 (100)	35 (100)	58 (100)	—
Intron 22 inversion	57 (50.4)	14 (60.9)	43 (47.8)	18 (51.4)	27 (46.6)	
Nonsense mutation	28 (24.8)	2 (8.3)	26 (28.9)	7 (20)	19 (32.8)	
Large deletion or insertion	15 (13.3)	2 (8.3)	13 (14.4)	5 (14.3)	8 (13.8)	
Splice-site mutation	6 (5.3)	2 (8.3)	4 (4.4)	2 (5.7)	2 (3.4)	
Frameshift mutation	3 (2.7)	1 (4.2)	2 (2.2)	1 (2.9)	2 (3.4)	
Intron 1 inversion	2 (1.8)	0	2 (2.2)	2 (5.7)	0	
Unknown	2 (1.8)	2 (15.4)	0	0	0	
Follow-up period (from ITI/EMI treatment-start to data analysis) (y)	2.5 (2.2)	3.7 (3.1)	2.4 (2.3)	1.4 (0.9)	3.4 (1.8)	—

BU, Bethesda units; EMI, emicizumab; ITI, immune tolerance induction; MD-ITI, intermediate-dose immune tolerance induction group (factor [F]VIII, 100 IU/kg/d); LD-ITI, low-dose immune tolerance induction (FVIII 50 IU/kg every-other-day).

a The *P* value indicates the comparison between EMI prophylaxis group and total-ITI group.

b The proportion was calculated using included patients who had received genetic testing as the reference cohort.

In total, 49 parents/guardians completed parent proxy-reported rating for the CHO-KLAT at baseline. In this group, 21 children were 7 years of age or older, and thus provided self-reports. The median baseline CHO-KLAT score reported by 49 parents/guardians was 55.0 in EMI group, 57.5 in MD-ITI group, and 56.0 in LD-ITI group. The median baseline CHO-KLAT score reported by 21 children was 58.0 in EMI group, 57.0 in MD-ITI group, and 58.0 in LD-ITI group.

In total, 113 enrolled patients underwent FVIII gene testing, and the intron 22 inversion had the largest proportion, accounting for 50.4%, followed by 24.8% for nonsense mutation and 13.3% for large deletion or insertion (Table 1).

### 3.2 Comparison of bleeding control between EMI group and ITI group

#### 3.2.1 ABR and AJBR

Bleeding rates by different treatment regimens and different observation periods are shown in Figure A, B and Table 2. EMI prophylaxis demonstrated significantly lower bleeding rates across all study phases, with median ABR and AJBR consistently at 0, significantly lower than those in the ITI group (*P* < .001). With prolonged treatment, bleeding rates in the ITI group progressively declined and the therapeutic advantage of EMI over ITI in bleeding management progressively decreased. Within the ITI cohort, the MD-ITI group showed significantly better bleeding-control outcomes than the LD-ITI group. Notably, no life-threatening bleeding events were documented in any of the 3 treatment groups throughout the study duration.

**Figure:**
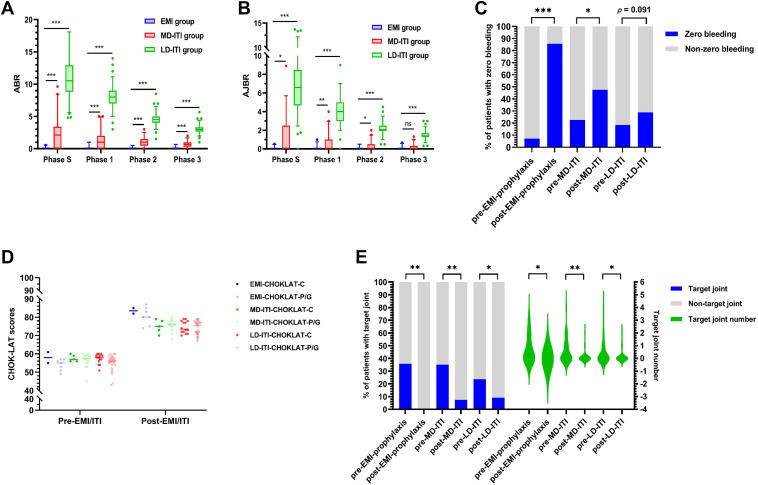
(A) The ABR among EMI group, MD-ITI group and LD-ITI group by different treatment period. (B) The AJBR among EMI group, MD-ITI group, and LD-ITI group by different treatment period. (C) The proportion of zero bleeding. (D) The CHO-KLAT scores at baseline and after EMI/ITI treatment. (E) The proportion and number of target joint. Phase S (success-equivalent phase): for the ITI group: duration from treatment initiation to achievement of ITI success (defined as 2 consecutive negative inhibitor titers of ≥4 weeks apart with FVIII recovery of ≥66% of expected); for the EMI group, the average time to ITI success, derived from our ITI cohort (mean, 9.5 months), ensuring comparable observation windows; phase 1: 0-12 months (first year from treatment initiation); phase 2: 0-24 months (first 2 years from treatment initiation); phase 3: 0-36 months (first 3 years from treatment initiation). ∗*P* < .05; ∗∗*P* < .01; ∗∗∗*P* < .001. ABR, annualized bleeding rate; AJBR, annualized joint bleeding rate; CHO-KLAT, Canadian Hemophilia Outcomes-Kids Life Assessment Tool; EMI, emicizumab; EMI-CHOKLAT-C, the self-reports of children were 7 years of age or older in EMI group; EMI-CHOKLAT-P/G, parent proxy-reported rating of the parents/guardians in EMI group; ITI, immune tolerance induction; LD-ITI, low-dose immune tolerance induction (FVIII, 50 IU/kg every-other-day); LD-ITI-CHOKLAT-C, the self-reports of children were 7 years of age or older in LD-ITI group; LD-ITI -CHOKLAT-P/G, parent proxy-reported rating of the parents/guardians in LD-ITI group; MD-ITI, intermediate-dose immune tolerance induction group (factor [F]VIII, 100 IU/kg/d); MD-ITI-CHOKLAT-C, the self-reports of children were 7 years of age or older in MD-ITI group; MD-ITI-CHOKLAT-P/G, parent proxy-reported rating of the parents/guardians in MD-ITI group.

**Table 2:** The bleeding rates by treatment arm and observation period.

		EMI	MD-ITI	*P*	LD-ITI	*P*
Phase S						
ABR	Median (IQR)	0 (0)	2.1 (3.4)	<.001	10.6 (4.0)	<.001
	Range^a^	0-0.56	0-13.6		4.8-29.6	
AJBR	Median (IQR)	0 (0)	0 (2.5)	.021	6.7 (3.8)	<.001
	Range^a^	0-0.54	0-10.9		0-13.7	
Phase 1						
ABR	Median (IQR)	0 (0)	1.0 (2.0)	<.001	8.0 (2.0)	<.001
	Range^a^	0-1.0	0-5.0		5.0-14.0	
AJBR	Median (IQR)	0 (0)	0 (1.0)	.009	4.0 (2.0)	<.001
	Range^a^	0-1.0	0-4.0		0-9.0	
Phase 2						
ABR	Median (IQR)	0 (0)	1.0 (1.0)	<.001	4.5 (1.0)	<.001
	Range^a^	0-0.5	0-3.0		2.5-8.5	
AJBR	Median (IQR)	0 (0)	0 (0.5)	.019	2.0 (0.5)	<.001
	Range^a^	0-0.5	0-2.0		0.5-4.5	
Phase 3						
ABR	Median (IQR)	0 (0)	0.7 (0.7)	<.001	3.0 (0.9)	<.001
	Range^a^	0-0.7	0-2.0		1.7-5.7	
AJBR	Median (IQR)	0 (0)	0 (0.3)	.058	1.3 (0.3)	<.001
	Range^a^	0-0.7	0-1.3		0.3-3.0	

Phase S (success-equivalent phase): for the ITI group, duration from treatment initiation to achievement of ITI success (defined as 2 consecutive negative inhibitor titers ≥4 wk apart with FVIII recovery of ≥66% of expected); for the EMI group, the average time to ITI success, derived from our ITI cohort (mean, 9.5 months), ensuring comparable observation windows; phase 1: 0-12 months (first year from treatment initiation); phase 2: 0-24 months (first 2 years from treatment initiation); phase 3: 0-36 months (first 3 years from treatment initiation).

ABR, annualized bleeding rate; AJBR, annualized joint bleeding rate; EMI, emicizumab; ITI, immune tolerance induction; LD-ITI, low-dose immune tolerance induction (FVIII, 50 IU/kg every-other-day); MD-ITI, intermediate-dose immune tolerance induction group (factor [F]VIII, 100 IU/kg/d).

a Range, minimum to maximum.

#### 3.2.2 Target joints

The target joints were documented in different treatment groups in Figure E. All treatment groups (EMI/MD-ITI/LD-ITI) witnessed a significantly decreased in proportion of target joints from baseline to post-EMI/ITI treatment (EMI group—35.7% vs 0; *P* = .002; MD-ITI group—35% vs 7.5%; *P* = .003; LD-ITI group—23.7% vs 9.2%; *P* = .014). The EMI group had the largest reduction proportion of target joints. The lowest reduction rate was observed in the LD-ITI group.

#### 3.2.3 Zero bleeding proportion

Both EMI group (85.7% vs 7.1%; *P* < .001) and MD-ITI group (47.5% vs 22.5%; *P* = .031) showed a significant increase in ZBP after treatment compared with baseline. Additionally, higher increased trend was observed in EMI group compared with MD-ITI group (78.6% vs 25%; *P* = .006). However, a numerical increasing trend in ZBP was documented in LD-ITI but without significant difference (28.9% vs 18.4%; *P* = .091) as shown in Figure C.

### 3.3 Comparison of QoL assessment between EMI group and ITI group

#### 3.3.1 CHO-KLAT scores

The parent proxy-reported and children self-reported CHO-KLAT scores after EMI or ITI treatment were shown in Figure D. All treatment groups witnessed a significant increase in CHO-KLAT scores (both parent proxy-reported and children self-reported). For children self-reported CHO-KLAT scores, compared with the LD-ITI group, the EMI group had significantly increased scores (25.5 vs 17.5; *P* = .013). Moreover, compared with MD-ITI group, the EMI group showed a greater increase, although with a boundary *P* value (25.5 vs 19.0; *P* = .060). As for parent proxy-reported CHO-KLAT scores, the highest increased was documented in the EMI group (EMI vs MD-ITI—26.6 vs 19.2; *P* < .001; EMI vs LD-ITI—26.6 vs 19.1; *P* = .002).

#### 3.3.2 Injection frequency

Compared with the MD-ITI or LD-ITI group, the EMI group had significantly lower mean infection frequency in phase S (EMI vs MD-ITI—3/mo vs 30/mo; *P* < .001; EMI vs LD-ITI—3/mo vs 15/mo; *P* < .001).

### 3.4 Comparison of medication cost between EMI group and ITI group

We performed a total medication cost analysis for the 3 treatment groups (EMI, MD-ITI, and LD-ITI) during phase S. This analysis included costs for both FVIII or EMI and bleeding control to characterize economic disparities in the inhibitor-positive phase.

Total medication cost in phase S for FVIII and bleeding control was similar between MD-ITI group and LD-ITI group (3703.4 vs 3656.3 US$/kg). However, the EMI group had significantly lower cost compared with MD-ITI or LD-ITI groups (1993.4 vs 3703.4 vs 3656.3 US$/kg; *P* < .001) (Table 3).

**Table 3:** The medication cost by treatment arm.

Variables, mean (SD)	EMI prophylaxis	MD-ITI	*P*^a^	LD-ITI	*P*^b^
EMI/FVIII cost (per kg; US$)	1985.5 (265.5)	3490.0 (1926.3)	<.001	2372.1 (1280.0)	.147
Cost for bleeding control (per kg; US$)	8.0 (16.8)	213.4 (304.1)	.002	1284.2 (854.1)	<.001
Total medication cost (EMI/FVIII + bleeding control; per kg; US$)	1993.4 (267.0)	3703.4 (2059.8)	<.001	3656.3 (2018.3)	<.001

Exchange rate: ¥7.2153 = US$ 1 (March 18, 2025).

EMI, emicizumab; ITI, immune tolerance induction; LD-ITI, low-dose immune tolerance induction (FVIII, 50 IU/kg every-other-day); MD-ITI, intermediate-dose immune tolerance induction group (factor [F]VIII, 100 IU/kg/d).

a The *P* value indicates the comparison between EMI prophylaxis and MD-ITI group.

b The *P* value indicates the comparison between EMI prophylaxis and LD-ITI group.

## 4 Discussion

This study provides the first comprehensive comparison of EMI prophylaxis vs ITI in SHAcwHTI, focusing on 3 critical clinical outcomes: bleeding control, QoL, and medication cost. Our findings demonstrate that EMI prophylaxis is associated with improved bleeding control (achieving median ABR and AJBR of 0), greater QoL improvement (as measured by CHO-KLAT scores), and significantly lower medication cost compared with ITI regimens, when evaluated without considering inhibitor eradication as an end point.

The fundamental treatment goal in hemophilia management—maintaining effective hemostasis to prevent bleeding complications and subsequent arthropathy [19]—remains significant regardless of inhibitor status. ITI aims to restore FVIII tolerance through intensive factor administration [20]. However, patients remain vulnerable to hemorrhages due to inhibitors, particularly in the early phase. Our data reveal that EMI provides more reliable bleeding protection throughout the treatment course, especially during this critical period. This clinical advantage is further magnified by EMI’s consistent efficacy regardless of inhibitor titer fluctuations, contrasting with ITI’s variable protection that depends on achieving immune tolerance.

Notably, our real-world Chinese cohort achieved satisfactory bleeding prevention (median ABR, 0; median AJBR, 0) with reduced-dose EMI regimens (median loading dose, 2.3 mg/kg/wk; maintaining dose, 5.4 mg/kg/mo), building upon previous reports of successful tailored EMI dosing prophylaxis (median loading dose, 3.0 mg/kg/wk; maintaining dose, 4.2 mg/kg/mo) in China [12]. These findings challenge the conventional dosage paradigm and suggest that tailored EMI dosing may maintain efficacy while improving accessibility in regions with constrained economy. The HAVEN 2 trial reported a median ABR of 0.3 and AJBR of 0.2 in participants aged <12 years receiving standardized EMI prophylaxis [21]. Notably, our study demonstrated numerically lower bleeding rates, with a median ABR and AJBR of 0, despite enrolling a younger patient population compared with that in HAVEN 2.

The QoL outcomes present further evidence for EMI’s clinical value. To our knowledge, this is the first documentation of CHO-KLAT score in SHAcwHTI. A post hoc analysis of the HAVEN 1, 3, and 4 and STASEY studies assessed the impact of EMI prophylaxis on pain-related QoL in persons with HA and found that pain-related QoL was improved [22]. A recent Chinese study (CHIPS) indicated that the HA without inhibitors receiving 4 years individualized low-dose prophylaxis achieved QoL improved, with mean CHO-KLAT scores ranging from 68.8 to 78.8. Notably, EMI-treated children and those with inhibitors in this study exceeded the QoL levels reported in CHIPS study, likely attributable to reduced treatment burden (fewer injections) and reliable bleeding protection.

Medication cost analyses revealed that EMI’s cost advantage (∼50% lower than ITI). Notably, our cost analysis represents a conservative estimate of EMI’s economic value. The actual cost-effectiveness is likely even greater, as we excluded substantial ancillary expenses specific to ITI, such as peripherally inserted central catheter, ISs, and hospitalizations. In resource-constrained settings where treatment cost plays a key role in therapeutic decision making, EMI’s combination of clinical effectiveness and economic efficiency makes it a priority. To validate the robustness of our economic findings, we performed a sensitivity analysis considering the impact of excluded ancillary costs. A scenario analysis indicates that if these ancillary costs were included, the total cost of the ITI cohort would increase substantially, while the EMI cohort—which is administered subcutaneously and is minimally resource intensive—would remain largely unaffected. Consequently, our reported cost difference represents a conservative estimate, and the superior cost-effectiveness of EMI is robust to the inclusion of these broader health care resource utilizations.

However, we acknowledge that EMI’s inability to eradicate inhibitors remains its fundamental limitation. While EMI showed the satisfactory bleeding control and manageable perioperative hemostasis in the STASEY trial with EMI [23], ITI retains unique value for inducing immune tolerance. Unlike EMI, which provides a functional cure for the bleeding phenotype, ITI offers the potential for an immunologic cure. Successful ITI confers unique long-term benefits that EMI cannot provide, including the following: (i) the potential resumption of FVIII replacement therapy, which is essential for achieving high peak factor levels during major surgery or severe trauma; (ii) the elimination of lifelong inhibitor-related complication risks and simplified emergency management; and (iii) the restoration of the body’s natural response to FVIII, offering a definitive resolution to the inhibitor status rather than lifelong reliance on nonfactor replacement therapy. This creates a complex risk–benefit balance where EMI excels in immediate clinical outcomes, while ITI offers potential long-term immunologic resolution—a decision that must be individualized based on patient characteristics and health care resources.

Our study has several limitations. First, its single-center retrospective design may limit generalizability. In China, the distribution of medical resources is extremely uneven. Therefore, patients with hemophilia from all over the country comes to our center for treatment, which can reduce the bias of single center. However, potential biases inherent to this design should be considered. Referral bias is possible, as our center is a national referral hub for complex pediatric hematology cases, which may attract patients with more severe disease, refractory inhibitors, or families with greater health-seeking motivation. Additionally, treatment selection could be influenced by unmeasured socioeconomic factors, family preferences, or clinician bias, particularly as EMI was not covered by national insurance during the study period. Consequently, these selection biases limit the causal interpretation of our findings, and the observed favorable outcomes of EMI should be viewed as an association within a real-world context rather than confirmatory evidence from a randomized trial. Second, the sample size of the EMI group (n = 24) is relatively small compared to the ITI group, reflecting the real-world accessibility issues. This imbalance may limit the statistical power of the comparisons. Not being covered by medical insurance restricts the use of EMI to some extent. Third, we acknowledge that defining phase S for the EMI group based on the average ITI duration (9.5 months) is an artificial alignment. While intended to provide a comparable observation window, this design choice may favor EMI, which achieves rapid steady-state hemostasis, and should be interpreted with caution. Fourth, we acknowledge the absence of multivariate and longitudinal modeling. Due to the limited sample size in the EMI group and the zero-inflated nature of the bleeding data (where EMI patients consistently maintained a median ABR of 0), complex regression models were prone to statistical instability. However, given that key baseline characteristics were well-balanced between groups (Table 1), we relied on robust nonparametric tests to ensure the validity of our comparisons. Fifth, QoL data (CHO-KLAT) were available only for a subset of patients (49 parent-proxy reports and 21 child self-reports). This limitation stems from the age applicability of the instrument, which requires children to be ≥4 years old for parent reporting and ≥7 years old for self-reporting. Consequently, QoL analysis was restricted to this age-eligible subcohort.

## 5 Conclusion

For SHAcwHTI, EMI prophylaxis showed favorable outcomes regarding bleeding control, QoL enhancement, and medication cost compared with ITI. These findings suggest that EMI prophylaxis is a valuable therapeutic option, particularly for patients prioritizing immediate bleeding control over inhibitor eradication or in settings where intensive ITI protocols are not feasible.
